# Supplementary material for: A Humanized Yeast Phenomic Model of Deoxycytidine Kinase to Predict Genetic Buffering of Nucleoside Analog Cytotoxicity
Source: Genes (Basel). 2019 Sep 30;10(10):770. doi: 10.3390/genes10100770 (PMC6826991; doi:10.3390/genes10100770)
Supplement: Supplementary file 1 [file genes-10-00770-s001.zip › Additional_Files/Additional_File7_GOTermHeatmaps.zip!/Additional_File7_GOTermHeatmaps/preribosome,_large_subunit_precursor.pdf]

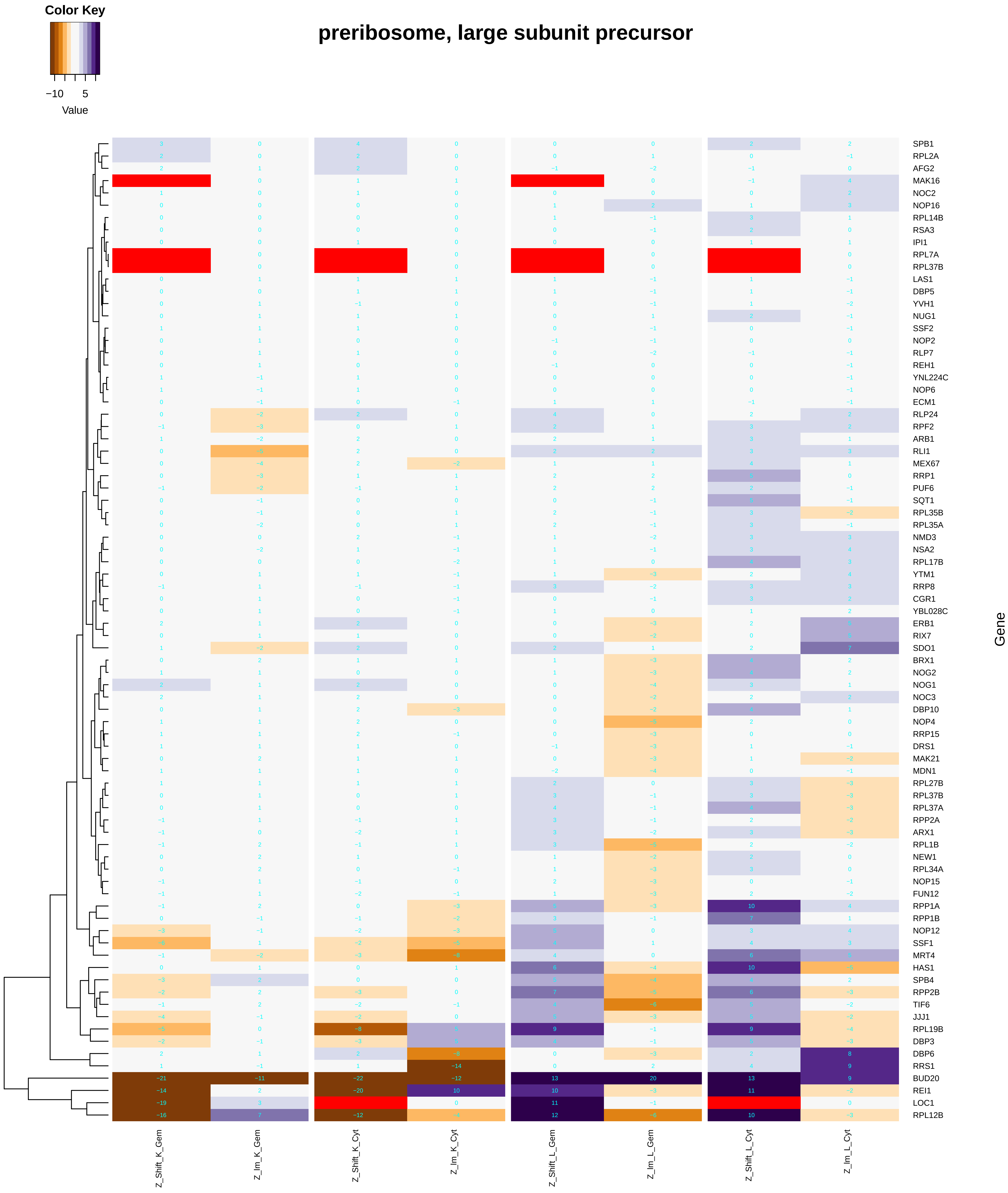
preribosome, large subunit precursor
Color Key
−10
5
Value
Gene
SPB1
RPL2A
AFG2
MAK16
NOC2
NOP16
RPL14B
RSA3
IPI1
RPL7A
RPL37B
LAS1
DBP5
YVH1
NUG1
SSF2
NOP2
RLP7
REH1
YNL224C
NOP6
ECM1
RLP24
RPF2
ARB1
RLI1
MEX67
RRP1
PUF6
SQT1
RPL35B
RPL35A
NMD3
NSA2
RPL17B
YTM1
RRP8
CGR1
YBL028C
ERB1
RIX7
SDO1
BRX1
NOG2
NOG1
NOC3
DBP10
NOP4
RRP15
DRS1
MAK21
MDN1
RPL27B
RPL37B
RPL37A
RPP2A
ARX1
RPL1B
NEW1
RPL34A
NOP15
FUN12
RPP1A
RPP1B
NOP12
SSF1
MRT4
HAS1
SPB4
RPP2B
TIF6
JJJ1
RPL19B
DBP3
DBP6
RRS1
BUD20
REI1
LOC1
RPL12B
Z_Shift_K_Gem
Z_Im_K_Gem
Z_Shift_K_Cyt
Z_Im_K_Cyt
Z_Shift_L_Gem
Z_Im_L_Gem
Z_Shift_L_Cyt
Z_Im_L_Cyt
